# Heart Rate Variability as Early Biomarker for the Evaluation of Diabetes Mellitus Progress

**DOI:** 10.1155/2016/8483537

**Published:** 2016-04-14

**Authors:** Rosa Elena Arroyo-Carmona, Ana Laura López-Serrano, Alondra Albarado-Ibañez, Francisca María Fabiola Mendoza-Lucero, David Medel-Cajica, Ruth Mery López-Mayorga, Julián Torres-Jácome

**Affiliations:** ^1^Escuela Superior de Medicina, Instituto Politécnico Nacional, Plan de San Luis y Díaz Mirón s/n, Colonia Casco de Santo Tomas, Delegación Miguel Hidalgo, 11340 Ciudad de México, DF, Mexico; ^2^Instituto de Fisiología, Benemérita Universidad Autónoma de Puebla, 14 Sur 6301, Colonia Jardines de San Manuel, 72570 Puebla, PUE, Mexico; ^3^Centro de las Ciencias de la Complejidad, Universidad Nacional Autónoma de México, Circuito Mario de la Cueva 20, Insurgentes Sur, Delegación Coyoacán, 04510 Ciudad de México, DF, Mexico; ^4^Ciencias Biológicas, Universidad Popular Autónoma del Estado de Puebla, 21 Sur 1103, Barrio de Santiago, 72410 Puebla, PUE, Mexico

## Abstract

According to the American Diabetes Association (ADA), the side effects of diabetes mellitus have recently increased the global health expenditure each year. Of these, the early diagnostic can contribute to the decrease on renal, cardiovascular, and nervous systems complications. However, the diagnostic criteria, which are commonly used, do not suggest the diabetes progress in the patient. In this study, the streptozotocin model in mice (cDM) was used as early diagnostic criterion to reduce the side effects related to the illness. The results showed some clinical signs similarly to five-year diabetes progress without renal injury, neuropathies, and cardiac neuropathy autonomic in the cDM-model. On the other hand, the electrocardiogram was used to determine alterations in heart rate and heart rate variability (HRV), using the Poincaré plot to quantify the HRV decrease in the cDM-model. Additionally, the SD1/SD2 ratio and ventricular arrhythmias showed increase without side effects of diabetes. Therefore, the use of HRV as an early biomarker contributes to evaluating diabetes mellitus complications from the diagnostic.

## 1. Introduction

The diabetes mellitus (DM) is considered like an important economic and social problem owing to the long-term complications such as premature death [1, 2]. Furthermore, the DM complications like neuropathies and failure renal increase the morbidity and cardiovascular mortality [3]. For this reason, the global healthcare expenditure on complications rises each year [4].

The ADA recommended some medical and laboratory tests to diabetes diagnostic [5]; however, none of them is considered useful to evaluate the time course and damage caused by diabetes including renal and nervous systems injury and cardiovascular disease which are regarded as chronic diabetes mellitus (cDM) complications [2, 3, 6]. Therefore, it is necessary to determine if the DM is chronic or acute for the best diagnosis and prognosis of illness [7].

On the other hand, the study of the alterations on DM the murine family has been widely used because these animals are handy and susceptible to DM development. According to the literature, the DM models induced with a streptozotocin single dose (100–200 mg/kg i.p.) [8] showed mortality more than 20% in the first week after administration. Additionally, insulin administration over time is extremely necessary for animal's survival [9, 10].

The diabetic model showed different physiological alterations when insulin is administered [11]. For this reason, it is important to develop a DM model with longer time period survival, lack of insulin, and a noninvasive tool to determine an early diagnostic. The heart rate variability (HRV) was proved to be a noninvasive tool as valuable clinical evidence for the prognosis of cardiovascular events and several disorders [12, 13] although some studies revealed a lower HVR, associated with sudden death in humans [14, 15].

One way of the HRV measurement is done by the RR Poincaré plot, quantifying long (SD2) and short (SD1) term and SD1/SD2 (iHRV) ratio. These parameters could help prognosis in a variety of pathological entities [15, 16].

The aim of this study was to identify an early biomarker in clinical practices for the evaluation of DM progress as an adequate and effective technique, using a cDM-model without insulin administration.

## 2. Methods

### 2.1. Animal Model

Adult male mice CD1 of 8 weeks old with 33 g of weight in average were used. All the animals were maintained with a 12 : 12 h light-dark cycle (7:00–19:00) and allowed free access to pellets LabDiet 5001 and water. All the experiments conducted on mice were approved by the Animal Care Committee of the Instituto de Fisiología Celular, Universidad Nacional Autónoma de México. Animal care was supported by the “International Guiding Principles for Biomedical Research Involving Animals,” Council for International Organizations of Medical Sciences, 2013.

#### 2.1.1. Induction of Diabetes Mellitus

One week before induction of diabetes, oral rehydration salts (NaCl 3.5 g/L, KCl 1.5 g/L, and Na_3_C_6_H_5_O_7_ + H_2_O 2.9 g/L and glucose 20 g/L) were given to the mice, supplemented with 10% glucose to prevent fatal hypoglycemic [8].

The animals were kept in fasting for 4 hours before induction. The DM induction was a single injection of 120 mg/kg i.p. streptozotocin (STZ) (Sigma-Aldrich) [17]. The STZ was dissolved with 0.12 mL of isotonic saline solution to pH = 7.4 instead of sodium citrate; during the preparation, the room remained in the darkness. This maneuver did not last more than 15 minutes [8].

After administration, in the first week the mice consumed oral rehydration salts supplemented with 10% glucose. This allowed the lack of dehydration and hypoinsulinemic and hypovolemic shock in the process [6, 10]. In addition to the survival, oral rehydration salts were provided to the mice during the following nine weeks.

#### 2.1.2. Blood Collection and Clinical Chemistry

The mice were euthanized, 10 weeks after administration, due to the complications on electrical activity of heart, but not necessarily the vascular system [18]. The blood samples were obtained 1 minute before the euthanasia between 8:00 and 10:00 a.m., not maintaining animals fasting for metabolic parameters measurement. The glucose plasma, cholesterol high density lipoprotein cholesterol (HDL-c), and cholesterol quantification were determined by glucose oxidase, phosphotungstate, and cholesterol oxidase technical, respectively; lipoprotein lipase assays were used for triglycerides and chemiluminescence for insulin.

#### 2.1.3. The Metabolic Cage

After 9 weeks of administration, mice were placed in the metabolic cage for 24 hours (12 hours of light and 12 hours of darkness) to quantify urine, feces, water, and food intake. The visual exam and dipstick test were used for urinalysis (Combi sys plus screen 11; Analyticon). Previously, the mice were handled and placed in the metabolic cage for two hours daily during 5 days prior to test to avoid stress.

### 2.2. Chronic Diabetes Mellitus Evaluation: Electrocardiogram Record

Ten weeks after induction, the mice were anesthetized with pentobarbital sodium 0.63 g/kg i.p. and placed in supine position for recording ECG for 30 minutes. The bipolar ECGs were recorded with subcutaneous needle electrodes in the configuration lead I. The electrodes were placed in right and left in the fourth intercostal space. The ECG signal was amplified 700 times and filtered at 60 Hz. The signal was recorded on personal computer at sampling frequency 1 KHz and analyzed offline with Clamp Fit® program (Molecular Device).

The analysis of heart rate variability (HRV) was made with RR interval (RR_*i*_) time series. The ECG was recorded for thirty minutes and 100 values of RR_*i*_ were randomly chosen. The RR intervals were measured between consecutive beats [19]. Also, the QTc was calculated with the corrected Bazett formula [20]. All mice were continuously monitored to guarantee adequate ventilation and temperature.

### 2.3. Heart Rate Variability

To quantify the HRV time domain, the heart rate, SD1, SD2, and iHRV were calculated. The RR_i_ interval is the time between the maximum value of the QRS_*i*_ complex and the next maximum value of the QRS_*i*+1_ complex. The Poincaré plotted the RR_*i*+1_ interval as function of the previous RR_*i*_ interval. The heart rate is the inverse RR interval. SD1 is the standard deviation of the distances between all points of the Poincaré diagram and RR_*i*+1_ = RR_*i*_ line. SD2 is the standard deviation of the distance between all points of the Poincaré diagram and ${RR}_{i+1}=-{RR}_{i}+2{\overset{-}{RR}}_{i}$ line where ${\overset{-}{RR}}_{i}$ is the average value of all RR_*i*_ [16]. iHRV is the SD1/SD2 ratio which is the value, thus suggesting the delicate equilibrium between sympathetic and parasympathetic systems on heart [14, 21–23].

### 2.4. Data Analysis and Statistics

All data is presented as the mean ± standard error. The *t*-test was used for data analysis; the values were considered statistically significant if value was lower than 0.05 denoted by *∗*. The analysis was done with program Origin Pro version 8.0 Lab Corporation.

The distances for obtained SD1 and SD2 were calculated with next equations:

(1) $$\begin{array}{c} \sqrt{{\left(\frac{{RR}_{i}-{RR}_{i+1}}{2}\right)}^{2}}, \end{array}$$

(2) $$\begin{array}{c} \sqrt{2{\left(\frac{{2\overset{-}{RR}}_{i}-{RR}_{i}-{RR}_{i+1}}{2}\right)}^{2}}. \end{array}$$

With all distances (1) and (2) equations, the SD1 and SD2 standard deviations were determined, respectively.

## 3. Results

### 3.1. Characterization of Diabetes Mellitus Chronic Model

**Figure 1:**
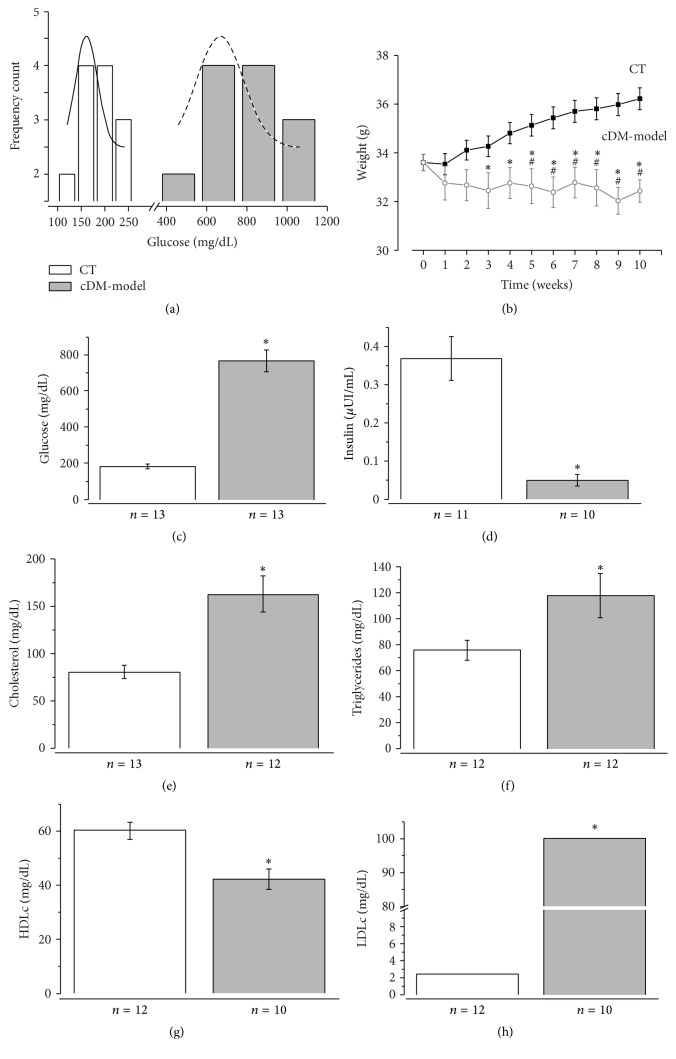
Characterization of chronic diabetes mellitus model. (a) Distributions of glucose plasma. (b) The plot shows that the cDM-model mice are losing weight since the third week after STZ-injection. (c) Increased plasma glucose. (d) Decreased insulin plasma. (e–h) Nonfasting lipid profile (LDL, HDL, total cholesterol, and triglycerides) is altered for the treatment. *n* = animals number, ^*∗*^ *p* (<0.05) versus control (dark line), and #: versus first weight (grey line).

In this research, 22 mice were used for cDM-model and 20 for control. All the injected animals had glycosuria, ensuing seven days after induction with mortality of 10%. Also, the control animals showed a growth on weight from 35 ± 0.5 g to 39.5 ± 0.7 g in the following 10 weeks after the injection while the cDM-model presented lowering weight at 33.6 ± 0.7 g in the last week. The loss of weight was evident from third week after injection of STZ (Figure 1(b)). In both conditions, after the tenth week of injection, the plasma glucose was measured; all the obtained values showed two normal distributions and were characterized by a mean of 161.6 ± 46.8 mg/dL and 730 ± 123.2 mg/dL (fitting results). The first normal distribution corresponds to the glucose of the control, and the second normal distribution is the glucose of injected animals. Consequently, the concentration of control glucose is considered if the values were from 68.1 to 255.1 mg/dL whereas the values of glucose in the diabetic were from 483.6 to 976.4 mg/mL (cDM-model). If the value of glucose was more than the control but less than the diabetic, the mouse was regarded hyperglycemic, not yet diabetic (Figure 1(a)).

The cDM-model had plasma glucose of 769 ± 216 mg/dL; hence the animals were diabetic. These had an insulin decrease of 7-fold, considering dyslipidemia like in humans. The cholesterol had 81.7 mg/dL and triglycerides 76 ± 8 mg/dL in the control, and in cDM-model 163 ± 19 mg/dL cholesterol and 118 ± 17 mg/dL triglycerides were presented. The HDL-c lipoprotein decreased by 39% in cDM-model and the LDL values was obtained by Friedewald formula because it is theoretical [24]. This value in control was 2.4 mg/dL and 100 mg/dL in cDM-model (Figure 1).

### 3.2. Metabolic Cage

In cDM-model, the volume of water intake had a 10-fold increase as excreted urine volume; the food intake grew by 60% and defecated by 107% more than control (Table 1). The urinalysis showed that both groups had clear urine. Furthermore, the dipstick tests showed glycosuria and increase in the blood, ketones, proteins, nitrites, and leukocytes in the cDM-model (Table 1).

### 3.3. Evaluation Progress DM: Electrocardiogram

**Figure 2:**
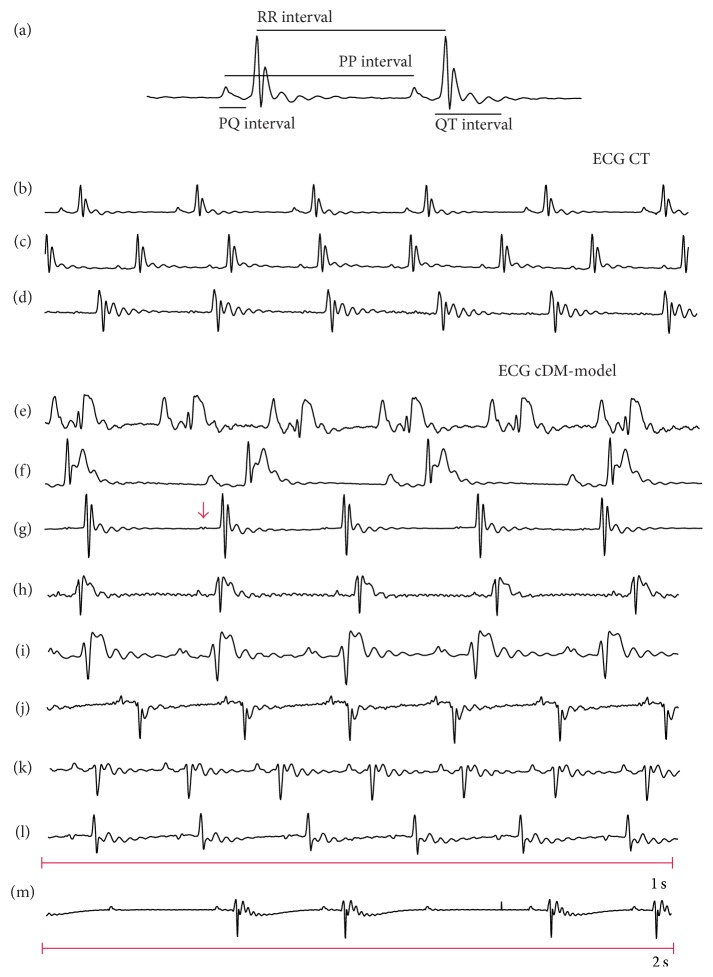
Electrocardiogram recording. (a) ECG intervals (b, c, d) show ECG control conditions. (e–m) ECG from cDM-model. (e, g, h) and (i) have an elevated ST segment. (j) and (k) have a depression QRS complex. (l) P-wave inverted. (g) The arrow show p-notched. (m) Second-degree block. The segments have 1 second of record except in (l) have 2 seconds because block produced intense bradycardia.

The HR was not affected by the diabetes mellitus because the HR in control mice had 482 ± 5 bpm and cDM-model had 488 ± 5 bpm (*p* < 0.05). However, 42% of diabetic mice exhibited supraventricular arrhythmias (8% of inversion in P-wave and 33% of P-notched). The 67% of diabetic mice showed ventricular arrhythmias like 50% QRS-complex inversion and 33% T-wave height of the animals. Also, 8% decrease of both T-wave and QRS-interval amplitude was shown, presenting 8% block of second-degree arrhythmia (Figure 2).

Other studies showed sympathetic and parasympathetic modulation on heart rate, the influence of nervous system on frequency and variation (SD1, SD2 and SD1/SD2 ratio) was altered from one disease to another, and an aging process [25, 26]. In this case, a Poincaré plot was used to establish the HRV for diabetic chronic. The alterations presented, matching in 3–5 stages of chronic disease which are associated with accelerated cardiovascular diseases like coronary disease [27].

**Figure 3:**
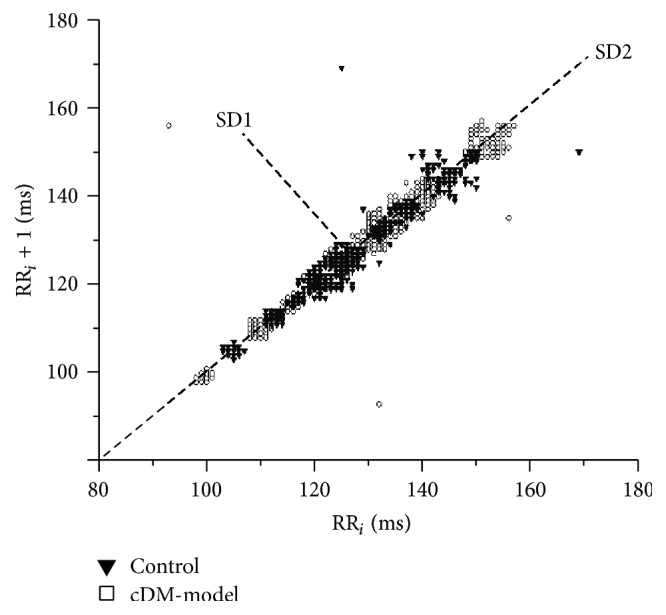
The Poincaré plots. The plot was constructed with RR interval showing an increase in SD2 in diabetic animals. The open square shows values from cDM-model and control in dark triangle.

The diabetic mouse had a decrease in the HRV (Figure 3). In control, SD1 was 1 and SD2 was 1.3; in diabetics SD1 was 0.9 and SD2 was 0.8. The iHRV was increased from 0.8 to 1.1 in cDM-model (Table 2). The heart rate of the control before and after administration was 275 ± 86 bpm with iHRV of 0.8.

The SD1 and SD2 parameters before the STZ-injection (time 0) were SD1 = 14 and SD2 = 18; after ten weeks they reduced to SD1 = 1 and SD2 = 1.3; however, the iHRV value remained constant (0.8), implying the nonexistence of changes in the delicate balance between sympathetic and parasympathetic systems by aging [23, 28].

Other reports associate the QTc prolongation and vascular diseases with risk factors of 92% increase in mortality and decrease the survival in the forthcoming 8 years in diabetic patients [29]. In cDM, the RR interval did not change; however, the QT interval increased by 11%, comparing with control. In addition, the QTc was prolonged to 17% (Table 2) without vascular troubles because the ratio heart mass/body weight was similar (control *n* = 18, 80 ± 2.6  × 10^−4^; cDM *n* = 15, 84 ± 4.3 × 10^−4^).

## 4. Discussion

In the literature, the STZ-models are considered diabetic when the plasma glucose was greater than 270 mg/dL [8, 10]; using the suggestions of the ADA to evaluate the diabetic animals, the mice showed dyslipidemias and impaired plasma glucose [30]. Additionally, our model presented erythrocytes and leucocytes with the absence of the bilirubin and urobilinogen (Table 1) in the urine test. The results demonstrated chronic diabetes in the cDM with an infection in the urinary tract without injury on the vascular, renal, and hepatic systems. These signs presented some similarities to chronic diabetes patient in second stage, developed in kidney disease due to the fact that these animals had proteins and blood in urine. The DM patients, who showed proteins and blood in urine, should have approximately from five to ten years of diabetes [31]. These conditions supported chronic DM, and these individuals usually showed kidney failure, in the ensuing 15 to 25 years [32].

In this proposal, the cDM-model has polyuria, polydipsia, and polyphagia, clinical signs which are exhibited in the chronic diabetic patients [30]. The diabetic animals did not present arrhythmias by dehydration in plasma because the electrolytes did not change in the cDM (Table 1).

Previous studies have shown that the dynamic HRV is a cut-off point to imbalance the nervous system on renal, cardiovascular, and endocrinology systems [22]. The R-R fluctuations allowed identifying some alterations in those systems. However, recent investigations showed that the HRV data are associated with the healthy subjects or chronic disease with side effects. Thus, in our model, the SD1 and SD2 alterations were determined without side effects (see above).

Additionally, the cDM-model showed variability in the dynamic RR interval which was associated with supra- and ventricular arrhythmias (Figure 3). In Poincaré plot of the QT segment plot, an increase was observed in SD2, related to lethal ventricular arrhythmias. Loss on the activity of autonomic system is shown. The long-term variability or SD2 was lower in 48% and the Poincaré (SD1/SD2) index was 38% higher than control. Our study implied that the animals could be in early stages of the nephropathy [33, 34], besides ventricular arrhythmias.

## 5. Conclusion

In summary, in our cDM-model, ventricular arrhythmias were shown, associated with long-term QTc, causing the increase of comorbidity and sudden death [35]. These arrhythmias are correlated to the potassium currents alterations in mice with the same treatment [11]. The changes on the total current of the membrane were associated likewise with alterations in the iHRV [36].

Further, the heart rate did not change in cDM, suggesting that diabetes, in this step, did not present even cardiac autonomic neuropathy [37] because of the iHRV remaining with the influence of sympathetic and parasympathetic tones [38]. This research demonstrated that the first damage was caused on cardiac electrophysiology by diabetes, before neuropathies and nephropathies. The electrophysiological changes in the cDM-model were demonstrated to be independent of vascular, infectious processes and disturbances in electrolytes. The current analysis proposed that SD1 and SD2 and SD1/SD2 ratio are early biomarkers for evaluating the progress of diabetes.

## Figures and Tables

**Table 1:** Characterization of diabetes in cDM-model.

Metabolic parameters		
	CT (*n* = 33)	cDM-model (*n* = 21)
Water intake (mL)	6 ± 0.4	60 ± 7^*∗*^
Food intake (g)	5 ± 0.2	8 ± 0.7^*∗*^
Feces excreted (g)	1.8 ± 0.2	5 ± 0.5^*∗*^
Urine excreted (mL)	0.4 ± 0.1	40 ± 6^*∗*^

Clinical biochemistry values		
Serum electrolytes	CT (*n* = 5)	cDM-model (*n* = 6)

Sodium (mmol/L)	149 ± 1	154 ± 4
Potassium (mmol/L)	9 ± 0.5	11 ± 0.4
Chloride (mmol/L)	115 ± 2	115 ± 4
Calcium (mg/dL)	9.4 ± 0.2	9.5 ± 0.6

Urine test strip		
	CT (*n* = 21)	cDM-model (*n* = 20)

Ketones	Negative (100%)	0.1 ± 0.06 (20%)
Glucose (mg/dL)	Negative (100%)	722.5 ± 149.7 (100%)
Protein (mg/dL)	Traces (47%)	24 ± 6 (85%)
Blood (ery/*µ*L)	Negative (100%)	16.1 ± 4.54 (50%)
pH	6.40 ± 0.14 (100%)	6.05 ± 0.2 (100%)
Nitrites	Positive (9%)	Positive (43%)
Leukocytes (leuk/*µ*L)	Negative (100%)	30.83 ± 8 (30%)
Specific gravity	1.02 ± 0.001 (100%)	1.01 ± 0.0006 (100%)
Urobilinogen	Negative (100%)	Negative (100%)
Bilirubin	Negative (100%)	Negative (100%)

The data obtained on metabolic cage and urine of 24 hours. The values are described as mean ± SEM. ^*∗*^Student's *t*-test  *p* < 0.05.

**Table 2:** Heart rate variability.

Interval (ms)	SD1	SD2	Poincaré index	
			SD1/SD2	Variability
RR				
Control = 126 ± 1.6	1	1.3	0.8	SD1 10%; SD2 39%
cDM-model = 125 ± 1.2	0.9^*∗*^	0.8^*∗*^	1.1^*∞*^	SD1/SD2 38%
QT				
Control = 39.3 ± 1.1	2	3.5	0.6	SD1 60%
cDM-model = 45.3 ± 0.8^∞^	0.8^*∗*^	3.4	0.2^*∗*^	SD1/SD2 60%
QTc				
Control = 35 ± 1.6	0.03	0.2	0.15	SD2 300%
cDM-model = 41 ± 0.7^*∞*^	0.03	0.6^*∞*^	0.05^*∗*^	SD1/SD2 33%

Control, *n* = 10; cDM-model, *n* = 21. Control ^*∗*^decrease, ^*∞*^increase; Student's *t*-test  *p* < 0.05.

## Additional Points

Clinical implications of this study are as follows. Consistent with our recent study findings, a noninvasive, straightforward, and inexpensive method was developed. On the other hand, this data treatment only requires arithmetic calculus, being a better procedure of DM chronic diagnostic and health care, preventing side effects in DM patients.
